# Increased serum levels of TGFβ1 in children with localized scleroderma

**DOI:** 10.1186/1546-0096-5-22

**Published:** 2007-12-03

**Authors:** Yosef Uziel, Brian M Feldman, Bernice R Krafchik, Ronald M Laxer, Rae SM Yeung

**Affiliations:** 1 Meir Medical Center, Kfar Saba, Tel Aviv University, Israel; 2 Divisions of Rheumatology, Department of Pediatrics, The Hospital for Sick Children, Toronto, Canada; 3 Departments of HPME and PHS, University of Toronto, Toronto, Canada; 4 Divisions Dermatology, Department of Pediatrics, The Hospital for Sick Children, Toronto, Canada; 5 Departments of Immunology and Medical Sciences, University of Toronto, Toronto, Canada

## Abstract

**Background:**

There are neither sensitive nor specific laboratory tests for measuring disease activity in localized scleroderma (LS). Monitoring is done almost exclusively by clinical assessment. Our aim was to determine whether serum concentrations of TGFβ1 are a good biomarker of disease activity in children with LS.

**Methods:**

55 pediatric patients with LS were divided into sub-types according to their main lesion; morphea, generalized morphea, linear scleoderma affecting a limb or the face. The lesions were further categorized by overall clinical assessment into active, inactive, and indeterminate groups according to disease activity. Serum TGFβ1 concentration levels were measured by enzyme linked immunosorbent assay (ELISA), analyzed and correlated with disease subtypes and disease activity.

**Results:**

The mean TGFβ1 concentration were significantly higher in the patient group (51393 ± 33953 pg/ml) than in the control group (9825 ± 5287 pg/ml) (P < 0.001). The mean concentration were elevated in all the disease subtypes, and did not correlate with disease duration or activity.

**Conclusion:**

Serum concentration of TGFβ1 were elevated in patients with all subtypes of LS irrespective of clinical disease activity. Although TGFβ1 may play an important role in the pathogenesis of local skin fibrosis, circulating blood levels of molecules known to act locally may not be useful biomarkers of disease activity.

## Background

Localized scleroderma (LS) is the most common type of scleroderma in children. LS differs from systemic sclerosis (SSc) by typically being confined to the skin and subcutaneous tissue, with only rare involvement of the internal organs [1,2]. There are neither sensitive nor specific laboratory tests for measuring disease activity, and monitoring is done almost exclusively by clinical assessment, which is often challenging. Identifying a laboratory marker of disease activity will aid in the management of affected children.

The pathogenesis of the skin fibrosis is far from clear; like other autoimmune diseases it is thought to involve an environmental trigger in an immuno-susceptible host leading to inflammation and damage, with increased production and deposition of collagen [3]. The Transforming Growth Factor β family of cytokines (TGFβ) plays a major role in modulation of the immune response, and is a critical counter-inflammatory/regulatory cytokine. TGFβ1 is also a central mediator in fibrosis and angiogenesis, playing an important role in the fibrotic process in scleroderma [3]. Several studies have found elevated serum TGFβ1 concentrations in both generalized and localized forms of sclerosis [4-6]. Skin TGFβ1 levels are more difficult to quantitate and in one study, no differences were found in affected skin from those with SSC [6] or LS [7]. Our study objectives were to determine the peripheral blood levels of TGFβ1 in children with LS and to determine the relationship of serum levels to disease activity.

## Patients and methods

### Patients

Serum samples were collected and cryo preserved from 55 patients who were evaluated at the LS clinic at The Hospital for Sick Children in Toronto. LS was diagnosed independently by both a board certified pediatric rheumatologist and a pediatric dermatologis, who run the multi-disciplinary clinic together. A consensus regarding diagnosis, disease subtype, and disease activity was jointly made. All clinical data were reviewed retrospectively from the patients' chart records. The patients were divided into four clinical subtypes according to their main lesion: Morphea (M), generalized morphea (GM) when they had a large area of the body involved and confluent or multiple morpheic lesions (3 or more lesions), or a linear band with 2 or more lesions, and linear scleroderma (LIN) on a limb (LIN limb) or on the face (LIN face "en coup de sabre"). The patients were further grouped according to their disease activity status which was determined clinically [1]. "Active phase" of LS was defined as lesions growing in size, appearance of new lesions, or erythematous violaceous color; "non-active phase" as no new lesions and no change in size of previous lesions over a minimum 6 month period, "indeterminate" as those in whom it was unclear and difficult to determine clinically whether there was active skin inflammation or change from previous visit. Informed consent was obtained for study participation and the institutional Research Ethics Board approved the study.

### Assays for TGFβ1

Following informed consent, serum samples were taken at the time of clinical assessment. All samples were collected and processed within 1 hour of collection, aliquoted, and frozen at -800°C until time of assay. All samples were tested at the same time, and triplicate samples were run. Serum concentration of TGFβ1 (latent and active forms) were measured by enzyme linked immunosorbent assay (ELISA) (Medicorp, Montreal, Canada) according to manufacturer's protocol. Our laboratory control population consisted of randomly selected, age matched patients with atopic dermatitis, a non-fibrotic inflammatory skin condition selected as a specificity control, attending a concurrent dermatology clinic.

### Statistical analysis

Groups were compared for TGFβ1 serum concentrations using student t-test (for 2 group comparisons). Analysis of variance (ANOVA) was used when comparing 3 or more groups. Analyses were repeated using non-parametric statistics (Mann-Whitney U test corrected for ties, and Kruskal-Wallis analysis of variance respectively) – but as these analyses did not differ, likely because TGFβ1 was normally distributed, they are not reported. In order to assess whether TGFβ1 serum concentrations were related to length of disease and demographic factors, multiple regression models were developed using a stepwise approach. All analyses were carried out using Data Desk 6.2.l (Data Description, Ithaca, New York, 2003).

## Results

Fifty-five patients in various sub-type groups (table 1) were studied. The mean age (± SD) at disease onset was 9.2 ± 3.6 years, (range of 0.7 to 19.2 years, median 8.7 years). The mean disease duration at the time of TGFβ1 sampling was 4.9 ± 3.7 years.

**Table 1:** Patient sub-groups

Patient Group	Number of patients
M	10
GM	16
LIN face	11
LIN limb	18

**Figure 1:**
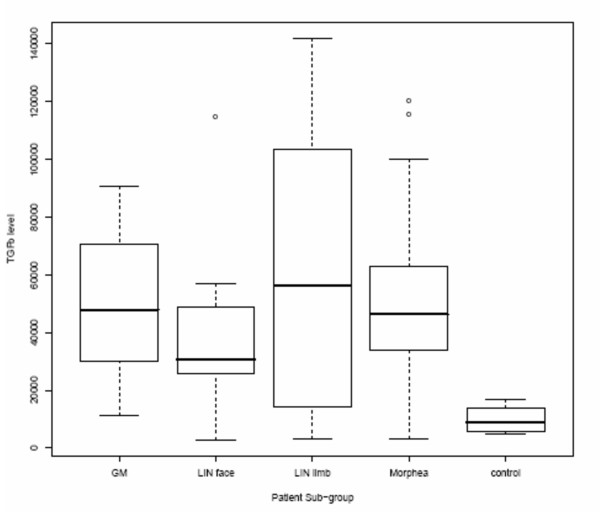
**TGFβ1 levels in LS and control groups**. Peripheral blood concentrations of TGFβ1 (pg/ml) in the different sub-groups of patients with LS and in controls. In the box and whisker plots, the horizontal line in the middle of the box represents the median, the bottom and top of the box represent one standard deviation from the mean, and the lines (whiskers) represent 2 standard deviations. Individual outlyers are represented by the circles above 2 SDs.

The mean serum TGFβ1 concentrations were significantly higher in patients than controls: 51393 ± 33953 pg/ml, (range 2949 – 141965 pg/ml), vs. 9825 ± 5287 pg/ml (range 4725 – 16761 pg/ml) (p < 0.0001). There was no significant difference between the TGFβ1 measurements between the 4 different subtypes as described above (Fig 1). (Mean TGFβ1 for LIN face = 39991 pg/ml, GM = 49053 pg/ml, M = 55,939 pg/ml, LIN limb = 58,481 pg/ml, F (3,51) = 0.714, p = 0.55.)

**Figure 2:**
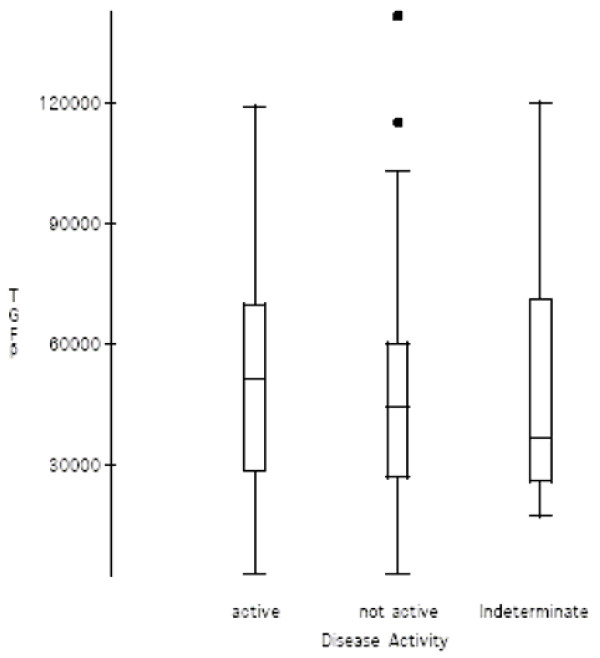
**TGFβ1 levels disease activity**. TGFβ1 serum concentrations (pg/ml) and disease activity. In the box and whisker plots, the horizontal line in the middle of the box represents the median, the bottom and top of the box represent one standard deviation from the mean, and the lines (whiskers) represent 2 standard deviations. Individual outlyers are represented by the filled squares above 2 SDs. Patient numbers: Active – 21 patients, Non active-23, Indeterminate-11.

There was no difference in TGFβ1 serum concentrations between the 21 patients in the active phase of the disease (51374 pg/ml), to the 23 patients with inactive disease (50985 pg/ml), or the 11 indeterminate phase patients (52284 pg/ml); F (2,52) = 0.0053, p = 0.99 (Fig. 2).

In a multiple regression model, there was no significant relationship between TGFβ1 serum concentrations and patient demographics (age, disease duration) or basic laboratory markers (ESR, eosinophil count, IgG concentrations or ANA),

## Discussion

Our study clearly demonstrates that serum concentratons of TGFβ1 are very elevated in pediatric LS patients at all times during evolution of their disease. The measurements were elevated in all the disease subtypes, and there was no correlation between the disease activity and the cytokine levels. This is in accord with previous studies that reported increased TGFβ1 serum concentrations in adults with SSc and LS [4-6].

TGFβ1 mediates fibrosis through downstream modulators. It is derived from many cellular sources including members of the immune system and those in other organ systems including activated endothelial cells. A critical downstream effect is stimulation of fibroblasts and excessive extra-cellular matrix deposition [3]. TGFβ1 is secreted as a pro-peptide (latent form) and enzymatically cleaved into the active form [8], and is a potent chemoattractant for human skin fibroblasts [9]. TGFβ1 also up-regulates synthesis of matrix metalloproteinase inhibitors, which inhibit collagenase activity [10].

In our study, there was no correlation between the serum concentrations of TGFβ1 and LS disease activity or disease subtype. There has been much work regarding an appropriate classification system for localized scleroderma. Interestingly, our data suggest that regardless of classification system used, this is a cohesive group of patients with elevation of circulating TGFβ1 as a common feature. Interestingly, high levels were also found in all phases of the disease included inactive states. Many different hypothesis can be invoked to explain this finding including modulation at the receptor level [11], as increased expression of TGFβ1 receptors are reported in skin fibroblasts of SSc and LS patients [12-14] and continued production of TGFβ1 by endothelial cells as a repair/wound healing response in inactive lesions. Alternatively, immune sources of TGFβ1 may be responsible for production of TGFβ1 as a regulatory/counter-inflammatory cytokine keeping the disease in the quiescent phase. Or it may simply by that peripheral blood levels of TGFβ1, a cytokine known to affect changes locally in only limited areas of affected skin, do not represent biochemical activity in affected skin; a scenario common to cytokines and enzymes which are tightly regulated at the tissue level. Another practical explanation of the lack of correlation between disease activity and TGFβ blood levels may be related to our limited clinical ability to determine disease activity accurately. It may be overly simplistic to assume a direct relationship between TGFβ1 and disease, as with other processes in the complex milieu of the human body, multiple mechanisms are at play to regulate the balance between extracellular matrix production and degradation.

Potential limitations of our study include population selection, specifically our control population. Our control group consisted of children with atopic dermatitis. LS is an inflammatory skin disease as is atopic dermatitis, thus making children with atopic dermatitis our choice for a specificity control for non- specific inflammation of the skin. Circulating levels of TGFβ1 in this population may be different from levels in healthy children. In fact, some reports suggest an association between low TGFβ1 responder genotype [15] and low TGFβ1 mRNA production by circulating leukocytes in some children with atopic dermatitis [16].

## Conclusion

Increased TGFβ1 serum concentrations appear to be common to all subtypes of children with LS suggesting a potentially useful role for TGFβ1 as a biomarker of the disease. Lack of correlation of circulating TGFβ1 concentration with disease activity preclude their use clinically to guide therapy in affected children.

## Competing interests

The author(s) declare that they have no competing interests.

## Authors' contributions

YU – participated in its design, and coordination of the study, and writing the manuscript.

BMF – participated in the design of the study and performed the statistical analysis, and helped to draft the manuscript.

BRK – participated in the design of the study, data collection, helped to draft the manuscript.

RML – participated in the design of the study, data collection, helped to draft the manuscript

RSMY -Senior author- participated in the design of the study, data collection and TGFβ1 analysis, and writing the manuscript.

All authors participated in writing, read and approved the final manuscript
